# Reason’s Triumph over Passion? Chinese Adults’ Attention to Information on Ultra-Processed Foods’ Fat and Sodium Contents in Nutrition Facts Tables

**DOI:** 10.3390/nu17010174

**Published:** 2025-01-02

**Authors:** Zeying Huang

**Affiliations:** Institute of Food and Nutrition Development, Ministry of Agriculture and Rural Affairs, Beijing 100081, China; huangzeying@caas.cn

**Keywords:** ultra-processed foods, fat, sodium, flavor labels, packaging

## Abstract

Background: It is common for consumers to purchase ultra-processed foods that are perceived to have health risks, and this phenomenon is rarely explained in the existing literature from the perspective of consumers’ responses to the intuitive marketing of flavor labels and the packaging. Methods: This study aimed to fill this knowledge gap and investigated the attention of 920 participants aged 18~59 across China toward fat and sodium content information for six ultra-processed foods (pastry foods, quick-frozen foods, dessert foods, puffed foods, beverages, and sauces) presented in nutrition facts tables based on the theoretical analysis framework for purchasing decisions on ultra-processed foods by using the binary logit model. Results: It was found that the respondent’ s attention to fat and sodium content information was positively influenced by health risk perception levels and levels of knowledge about fat and sodium but negatively influenced by the interaction term between flavor labels (or the packaging) that stimulated the purchase desire and health risk perception levels (or levels of knowledge about fat and sodium). Conclusions: The stimulation of purchase desire by flavor labels and the packaging weakened the consumer’ s increased attention to fat and sodium content information being enhanced by the health risk perception level and the level of knowledge about fat and sodium, especially the probability of attention to such information for dessert foods, puffed foods, quick-frozen foods, and sauces, which dropped the most. Additionally, the attention of females, youth, low-income individuals, those with below-college education, and non-overweight and obese individuals to such information dropped more, and the decrease was the largest for dessert foods, puffed foods, quick-frozen foods, and sauces.

## 1. Introduction

Healthier food selection using food labels such as nutrition facts tables (NFT) is one of the balanced diet guidelines advocated by The Dietary Guidelines for Chinese Residents (2022) [1]. Since 2013, food manufacturers in China have been mandated to label the energy, protein, fat, carbohydrate, and sodium contents per 100 g/mL of prepackaged food, along with their nutrient reference value percentages (NRV%), on the packages (Table 1). Ultra-processed foods (UPF) are formulations of food substances that are often modified using chemical processes and then assembled into ready-to consume hyper-palatable food and drink products using flavors, colors, emulsifiers, and a myriad of other cosmetic additives [2]. Common UPF include pastry foods, quick-frozen foods, dessert foods, puffed foods, beverages, and sauces. Among them, pastry foods such as bread, cake, cookies, and mooncakes are made with flour or rice flour, sugar, fat, eggs, dairy products, and various accessories, fillings, and seasonings through modulation, molding, cooking, and other processing [3]. Quick-frozen foods such as frozen pizza and frozen dumplings are rapidly processed at a low temperature (usually below −18 °C), with the addition of preservatives and flavors [4]. Dessert foods such as chocolate and ice cream are made with flour, sugar, egg, milk, and fruit through baking, steaming, and freezing [5]. Puffed foods such as potato chips and shrimp sticks are made from grains, tubers, beans, and vegetables through extrusion, expansion, drying, and seasoning [6]. Beverages such as milk tea, soda, and processed juice are made with added sugar, flavorings, colorings, and preservatives through preparation, sterilization, and filling [7]. Sauces such as salad dressings, mayonnaise, and ketchup are made with preservatives, colorings, and flavorings through preparation, boiling, filling, and sterilization [8]. Although UPF can meet consumers’ demands for convenience, taste, and appearance, regular over-consumption of these foods can lead to excessive intakes of sugar, fat, and sodium and a high incidence of obesity and diet-related chronic diseases [9]. Consumers are likely to reduce their purchases of UPF as NFT improve their understanding of fat and sodium contents. However, many consumers continue to purchase sugary drinks, chocolate, and cakes despite recognizing the health risks. Therefore, it is essential to explore the reasons behind the inconsistency between awareness and behaviors to effectively promote public nutrition education and improve residents’ dietary health. Our findings are expected to contribute valuable insights and implications to the global discourse on dietary health.

There are abundant studies examining the reasons behind the purchase of UPF by people with health risk perception and relevant knowledge on physiology, psychology, social connection, and nutrition claims. From the perspective of physiological addiction, UPF are delicious and convenient, which can make some consumers feel happy and dependent [10,11]. Some people believe that UPF help to relieve stress from life and work [12,13] and can reduce consumers’ anxiety or depression as they socialize with friends at parties, bars, and cafes to connect with others and integrate into social circles [14,15]. There is also confusion surrounding nutrition claims; some UPF labeled as high in protein, dietary fiber, and calcium, creates a “health halo” that can shift consumers’ negative attitudes toward UPF [16,17,18].

Intuitive marketing is a marketing strategy that emphasizes communicating with consumers in a direct, real, and interactive manner [19]. It can help consumers intuitively understand the actual situations of products or services, and enhance the brand images and sales of enterprises [20]. The intuitive marketing typically employed by ultra-processed food manufacturers, such as flavor labels and the packaging [21], can promote quick and emotional purchasing and dispel concerns about food health risks. However, this has yet to be studied. Therefore, this study attempted to fill the gap by investigating whether common UPF’s flavor labels and the packaging that stimulated Chinese consumers’ desire to buy interfered with attention to fat and sodium content information promoted by levels of health risk perception and knowledge about fat and sodium.

**Table 1:** An example of a nutrition facts table. Source: National Standard for Food Safety—General Rules for Nutrition Labelling of Prepackaged Food (GB 28050-2011) [22].

Items	Per 100 g	NRV (Nutrient Reference Value) %
Energy	1823 kJ	22
Protein	9.0 g	15
Fat	12.7 g	21
Carbohydrate	70.6 g	24
Sodium	204 mg	10

## 2. Theoretical Analysis Framework and Hypotheses

**Figure 1:**
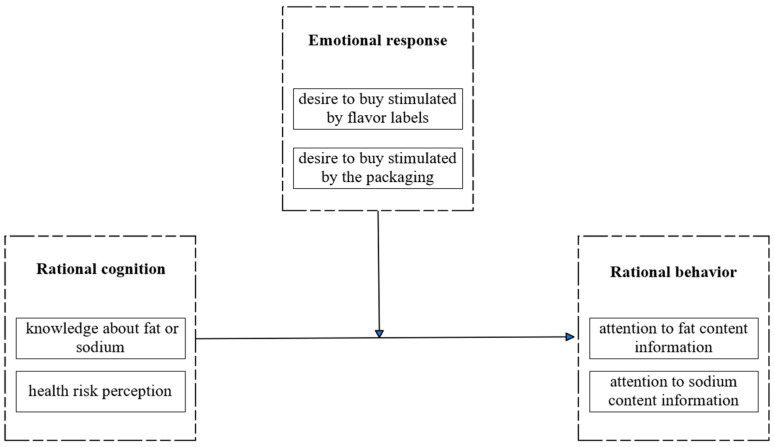
Theoretical analysis framework for ultra-processed food purchasing decision. Source: the author’s own work.

Dual process theory suggests that intuitiveness and reason, as two different human information-processing modes, are independent and complementary [23]. It is inferred that consumers’ food choices could be formed through the interaction of intuition and rational thinking processes. The theoretical framework of ultra-processed food purchase decisions proposed in Figure 1 illustrates that rational cognition boosts rational behavior, while emotional response interferes with rational cognition’s promotion of rational behavior. Specifically, rational cognition refers to consumers’ knowledge and correct cognition of products or services [24], such as knowledge about fat or sodium and the health risk perception of UPF. Emotional response refers to the pleasure and ease conveyed by the intuitive marketing of products or services to consumers [25], such as the stimulation of the desire to buy UPF by flavor labels and the packaging. Rational behavior is a deliberate behavior adopted by consumers [26], such as paying attention to fat and sodium content information of UPF.

Some findings showed that individuals with high levels of health risk perception regarding foods were likely to make purchase decisions based on nutrition labels [27,28]. In this regard, consumers were likely to pay attention to fat and sodium content information of UPF if they better understood that the excessive consumption of such foods could cause high blood fat and high blood pressure. Additionally, Knowledge–Attitude–Practice (KAP) theory emphasizes individuals’ knowledge as the basis of their behavior [29], and a high level of nutrition knowledge is positively related to the usage of food labels [30,31]. It is likely that consumers realize the importance of reasonable intakes of fat and sodium and pay attention to the information on such for UPF if they have good knowledge about these dietary factors.

Flavor labels provide information on foods’ flavor characteristics [32]. The labels not only help food manufacturers to establish brand images and improve their products’ market competitiveness, but also help the consumer to understand the foods’ tastes and aromas, and find the most suitable foods for his or her tastes [33]. Flavor labels have been proven to stimulate consumers’ appetites [34,35]. Most UPF contain complex additives to enhance the foods’ sensory qualities described by flavor labels [36]. The consumer stimulated by flavor labels to buy UPF may prioritize the expected flavor pleasure and hence reduce his or her attention to fat and sodium content information despite having high level of health risk perception and nutrition knowledge. Thus, Hypotheses 1, 2, 3, and 4 are proposed below:

**H1.**  *UPF’s flavor labels that stimulate the consumer’s desire to buy the foods, is likely to reduce the probability of attention he or she pays to fat content information raised by his or her health risk perception level.*

**H2.**  *UPF’s flavor labels that stimulate the consumer’s desire to buy the foods, is likely to reduce the probability of attention he or she pays to sodium content information raised by his or her health risk perception level.*

**H3.**  *UPF’s flavor labels that stimulate the consumer’s desire to buy the foods, is likely to reduce the probability of attention he or she pays to fat content information raised by his or her knowledge level about fat.*

**H4.**  *UPF’s flavor labels that stimulate the consumer’s desire to buy the foods, is likely to reduce the probability of attention he or she pays to sodium content information raised by his or her knowledge level about sodium.*

Product packaging is a general term for the use of containers, materials, and decorations to protect products, facilitate storage, and promote sales [37]. Food packaging design in line with the consumer psychology is likely to help manufacturers to establish brand images and win good reputations [38]. According to the visual communication design theory, product packaging, as a means of communication, is dedicated to attracting the consumer’s attention and diverting their focus from product attributes [39]. Relevant studies showed that the consumer’s food choices is mostly influenced by the colors, copy, shapes, and materials of food packages [40,41]. It is inferred that the consumer, even one with a high level of health risk perception and knowledge about fat and sodium, pays little attention to fat and sodium content information if his or her desire to buy UPF is stimulated by the package. Thus, Hypotheses 5, 6, 7, and 8 are proposed below:

**H5.**  *UPF’s packaging that stimulates the consumer’s desire to buy the foods, is likely to reduce the probability of attention he or she pays to fat content information raised by his or her health risk perception level.*

**H6.**  *UPF’s packaging that stimulates the consumer’s desire to buy the foods, is likely to reduce the probability of attention he or she pays to sodium content information raised by his or her health risk perception level.*

**H7.**  *UPF’s packaging that stimulates the consumer’s desire to buy the foods, is likely to reduce the probability of attention he or she pays to fat content information raised by his or her knowledge about fat level.*

**H8.**  *UPF’s packaging that stimulates the consumer’s desire to buy the foods, is likely to reduce the probability of attention he or she pays to sodium content information raised by his or her knowledge about sodium level.*

Studies showed that consumers who were female [42], younger [43], and with a low income [27,44], low education levels [45], and a healthy BMI [46] were more susceptible to intuitive marketing and focus on food sense but not quality. This is because women show more sensitive to certain types of information than men due to biological, social, and cultural differences [47]; young people tend to be more prone to impulse spending than middle-aged people due to differences in self-control tendencies [48]; low-income people may be less able to afford food that looks and tastes appealing and is of high nutritional quality [49]; people with low education levels may tend to have less nutritional knowledge [50]; people who are not overweight and obese do not have much need to lose weight and may therefore believe that they can freely choose foods [51]. Hence, these population groups are likely to reduce more attention to fat and sodium content information despite having health risk perception or knowledge about fat and sodium. Hypotheses 9, 10, 11, and 12 are proposed below:

**H9.**  *UPF’s flavor labels that stimulate the consumer’s desire to buy the foods, is likely to reduce more probability of attention female, youth, low-income, below college, and non-overweight and obese group pays to fat or sodium content information raised by his or her health risk perception level.*

**H10.**  *UPF’s flavor labels that stimulate the consumer’s desire to buy the foods, is likely to reduce more probability of attention female, youth, low-income, below college, and non-overweight and obese group pays to fat or sodium content information raised by his or her knowledge level about fat and sodium.*

**H11.**  *UPF’s packaging that stimulates the consumer’s desire to buy the foods, is likely to reduce more probability of attention female, youth, low-income, below college, and non-overweight and obese group pays to fat or sodium content information raised by his or her health risk perception level.*

**H12.**  *UPF’s packaging that stimulates the consumer’s desire to buy the foods, is likely to reduce more probability of attention female, youth, low-income, below college, and non-overweight and obese group pays to fat or sodium content information raised by his or her knowledge level about fat and sodium.*

## 3. Materials and Methods

### 3.1. Data Collection

The questionnaire was designed based on a literature review and expert consultation and contained 63 questions relating to demographic characteristics, nutrition knowledge levels, behavior toward nutrition facts tables, attitudes toward UPF’s flavor labels and the packaging, and health risk perception levels regarding UPF (see the Supplementary Material). Among them, each respondent’s level of knowledge about fat and sodium was tested using three objective questions, respectively. The respondent received 4 points for all questions answered correctly and 1 point for all questions answered wrong; a higher score indicates higher knowledge level. Each respondent’s health risk perception level for each UPF was tested using four subjective questions. The respondent received 5 points for all questions answered “yes” and 1 point for all questions answered “no”; a higher score indicates higher health risk perception level. Whether the respondent paid attention to fat or sodium content information of each UPF was tested using the self-reported question, and the question answered “yes” indicates that the respondent paid attention to the information. Whether flavor labels or the packaging stimulated the respondent’s desires to buy UPF was tested using five subjective questions, and all questions being answered “yes” indicated that the respondent’s desire to buy was stimulated. It should be noted that Cronbach’s α and Kappa values exceeded 0.8 and 0.6, respectively, which proved that the measurement items of whether respondent’s desire to buy was stimulated by flavor labels and the packaging, level of knowledge about sodium and fat, health risk perception levels all had high internal consistency reliability and content validity.

Data were collected through a paid online survey on Wenjuanxing (https://www.wjx.cn), a well-known online survey platform in China with a database of 6.2 million registered members of different ages. Firstly, the minimum sample size (N = 757) was determined based on an allowable error of 3% and a confidence level of 90%; then, a stratified sampling approach was employed to randomly select 30 participants aged 18 to 59 from each of China’s 31 provinces/autonomous regions from its member database. The quota random sampling method was adopted to obtain representative samples. Firstly, the Wenjuanxing member database was stratified according to gender (i.e., male or female), age (18~39 or 40~59 years old), education level (primary school or below, junior school, senior school, college, postgraduate, or above), and the annual household disposable income (CNY < 10,000, CNY 10,000~50,000, CNY 50,001~100,000, CNY 100,001~150,000, CNY 150,001~200,000, or CNY > 200,000) (i.e., USD < 1407, USD 1407~7033, USD 7034~14,067, USD 14,068~21,100, USD 21,101~28,133, or USD > 28,133 due to that USD 1 equals CNY 7.11 from 10 October to 25 October 2024) of each surveyed province, and the sample number was allocated based on the sample proportion of each layer. Then, the questionnaire link was emailed to randomly selected samples from each layer between 10 October and 25 October 2024. Prior to data collection, informed written consent was obtained from all the participants, who were informed they would receive CNY 8 as a cash incentive for providing accurate and complete responses. Finally, questionnaires continued to be sent until the sample quantity met the requirements. After data validity was checked, the responses from 920 participants were used for analysis.

### 3.2. Methods

Respondents’ attention to fat and sodium content information is expressed as a binary classification (1 = yes; 0 = no), so the binary logit model was used to conduct empirical analysis. The mathematical formula is expressed as follows.

(1) $$Y=\ln\left(\frac{p}{1-p}\right)=\alpha X\;+\;\varepsilon$$

where Y denotes a dummy variable for the respondent ’s attention to fat or sodium content information; p indicates the respondent’s attention probability; X represents a vector of control variables that influence the attention; α are the influence parameters; ε is a stochastic disturbance.

The influence of whether UPF’s flavor labels or the packaging that stimulates respondent i’s desire to buy affects his or her health risk perception levels regarding UPF and levels of knowledge about sodium or fat on whether he or she pays attention to fat or sodium content information is expressed as follows:

(2) $$\begin{array}{c} Y_{i}=\beta_{0}\;+\;\beta_{1}{Riskperception}_{i}\;+\;\beta_{2}{Knowledge}_{i}\;+\;\beta_{3}{Flavorlabelsorpackaging}_{i}\\ +\;\beta_{4}{Riskperception}_{i}\times {Flavorlabelsorpackaging}_{i}\;+\;{\;\beta }_{5}{Knowledge}_{i}\times {Flavorlabelsorpackaging}_{i}\;+\;\beta_{j}X_{ij}\;+\;\theta_{i} \end{array}$$

Formula (2) is used to test Hypotheses 1 to 8. $Y_{i}$ denotes a dummy variable for whether respondent i pays attention to fat or sodium content information; ${Riskperception}_{i}$ is a continuous variable for respondent i’s health risk perception levels; $\beta_{1}$ is the parameter of respondent i’s health risk perception level’s influence on whether he or she pays attention to fat or sodium content information; ${Knowledge}_{i}$ is a continuous variable representing respondent i’s level of knowledge about fat or sodium; $\beta_{2}$ is the parameter of the influence of respondent i’s level of knowledge about fat or sodium on whether he or she pays attention to fat or sodium content information; ${Flavorlabelsorpackaging}_{i}$ is a dummy variable representing whether flavor labels or the packaging stimulates respondent i’s desire to buy. $\beta_{3}$ is the parameter of the influence of whether flavor labels or the packaging stimulates respondent i’s desire to buy on whether he or she pays attention to fat or sodium content information; ${{Riskperception}_{i}}_{i}\times {Flavorlabelsorpackaging}_{i}$ is the interaction term between respondent i’s health risk perception level and whether flavor labels or the packaging stimulates his or her desire to buy; ${Knowledge}_{i}\times {Flavorlabelsorpackaging}_{i\;}$ is the interaction term between respondent i’s level of knowledge about fat or sodium and whether flavor labels or the packaging stimulates his or her desire to buy; $\beta_{1}{\;+\;\beta }_{4}$ is the interference effect of whether flavor labels or the packaging stimulates respondent i’s desire to buy regarding the influence of his or her health risk perception level on whether he or she pays attention to fat or sodium content information; $\beta_{2}{\;+\;\beta }_{5}$ is the interference effect of whether flavor labels or the packaging stimulates respondent i’s desire to buy regarding the influence of his or her level of knowledge about fat or sodium on whether he or she pays attention to fat or sodium content information; $\beta_{j}X_{i}$ represents a vector of control variables that influence whether respondent’s attention to fat or sodium content information. $\beta_{0}$, $\beta_{1}$, $\beta_{2}$, $\beta_{3}$, $\beta_{4}{,{\;\beta }_{5},\;\beta }_{j}$ are parameters to be estimated, and $\theta_{i}$ is a stochastic disturbance.

The role of whether UPF’s flavor labels or the packaging stimulates respondent i in group *k*’ s desire to buy, in influencing the effect of his or her health risk perception level and level of knowledge about sodium or fat on whether he or she pays attention to fat or sodium content information is expressed as:

(3) $$\begin{array}{c} Y_{ki}={\;\gamma }_{0}\;+\;{\;\gamma }_{1}{Riskperception}_{ki}\;+\;\gamma_{2}{Knowledge}_{ki}\;+\;\gamma_{3}{Flavorlabelsorpackaging}_{ki}\\ +\;\gamma_{4}{Riskperception}_{ki}\;\times \;{Flavorlabelsorpackaging}_{ki}\;+\;\gamma_{5}{Knowledge}_{ki}\;\times \;{Flavorlabelsorpackaging}_{ki}\;+\;\gamma_{j}X_{kj}\;+\;{\;\epsilon }_{ki}\; \end{array}$$

Formula (3) is used to test Hypotheses 9 to 12. $Y_{ki}$ denotes a dummy variable for whether respondent i in group *k* pays attention to fat or sodium content information; *k* indicates a certain group such as classification by gender (or age, income level, education level, BMI); $\gamma_{1}{\;+\;\gamma }_{4}$ is the interference effect of whether flavor labels or the packaging stimulates respondent i  in group *k*’ s desire to buy regarding the influence of his or her health risk perception level on whether he or she pays attention to fat or sodium content information; $\gamma_{2}{\;+\;\gamma }_{5}$ is the interference effect of whether flavor labels or the packaging stimulates respondent i  in group *k*’ s desire to buy regarding the influence of his or her level of knowledge about fat or sodium on whether he or she pays attention to fat or sodium content information. $\gamma_{j}X_{kj}$ represents a vector of control variables that influence the respondent i in group *k*’ s attention to fat or sodium content information. $\gamma_{0}$, $\gamma_{1}$, $\gamma_{2},$ $\gamma_{3}$, $\gamma_{4}{,{\;\gamma }_{5},\;\;\gamma }_{j}$ are parameters to be estimated, and $\epsilon_{ki}$ is a stochastic disturbance.

Given that the logit regression coefficients could only be used to judge the direction of independent variables’ influence on dependent variables, this study adopted the average marginal effect to obtain a partial derivative from the above binary logit model to calculate the interference effect of flavor labels and the packaging, is expressed as follows:

(4) $${\left.m\arg inal\;effect=\frac{\partial Prob(Y=n|X)}{\partial X}\right|}_{X=\bar{X}}(n=0,1)$$

where the marginal effect indicates the change in the probability of Y as X changes by 1 unit if the other variables are averaged.

## 4. Results

### 4.1. Descriptive Statistics

As shown in Table 2, the respondents were predominantly female (57.2%), were 35.5 years old on average, and mostly had a college education. Annual household disposable incomes of CNY 100,001 to 150,000 accounted for the majority of the participants, and the numbers of minors and elderly accounted for 0.24% and 0.18% regarding family size, respectively. A total of 27.6% suffered from chronic nutritional diseases. The average scores of the respondents’ knowledge levels about fat and sodium were 3.48 and 3.24, respectively. This indicates that the majority of the respondents had good understanding of the effects of fat and sodium on the human body, the maximum daily intakes, and the harm of frequent excessive intakes. The average scores of the respondents’ health risk perception levels regarding pastry foods, quick-frozen foods, dessert foods, puffed foods, beverages, and sauces were 3.98, 4.21, 3.77, 4.10, 4.41, and 3.36, respectively. Among them, the perception level of beverages was the highest, and that of sauces was the lowest. This shows that, compared with other UPF respondents were more likely to understand the health risks of obesity, hypertension, dyslipidemia, and type 2 diabetes caused by the excessive consumption of beverages, but not that of sauces. In total, 22.5%, 39.0%, and 60.2% believed that the NFT information was comprehensible, accurate, and authoritative; 82.0% and 63.2% indicated that UPF’s flavor labels and the packaging stimulated their desire to buy the foods.

### 4.2. The Interference Effect of Flavor Labels on Attention to Fat and Sodium Content Information

Stata (17.0, StataCorp LLC, College Station, TX, USA) [52] was used for logit regression analysis. The regression analysis, whose results are shown in Table 3, aimed to test Hypotheses 1 and 2, respectively, while the results in Table 4 are for Hypotheses 3 and 4. Those in Table 5 are for Hypotheses 5 and 6, respectively; those in Table 6 are for Hypotheses 7 and 8; those in Table 7 are for Hypotheses 9 and 10; those in Table 8 are for Hypotheses 11 and 12.

Table 3 describes that the respondent’s health risk perception level significantly posed positive influence on his or her attention to six UPF’s fat and sodium content information but the interaction term between flavor labels that stimulated the purchase desire and health risk perception level negatively affected the attention. As for the marginal effect, the probability of the respondent’s attention to the fat and sodium content information decreased with health risk perception level increased by 1 point and intervention of flavor labels that stimulated the purchase desire. Among them, the respondent’s attention to the fat content information of dessert foods decreased the most (by 9.6%), from 13.1% to 3.5% (13.1–9.6%). Also, attention to sodium content information of puffed foods decreased the most (by 10.8%), from 14.4% to 3.6% (14.4–10.8%).

Table 4 shows the positive effect of levels of knowledge about fat and sodium as well as the negative effect of the interaction term between flavor labels that stimulated the purchase desire and levels of knowledge about fat and sodium. As for the marginal effect, the probability of attention the respondent paid to fat and sodium content information decreased due to joint influence of levels of knowledge about fat and sodium increased by 1 point and flavor labels that stimulated the purchase desire, especially attention to fat content information of puffed foods decreased the most (by 17.6%), from 20.5% to 2.9% (20.5–17.6%) while attention to sodium content information of quick-frozen foods decreased the most (by 27.4%), from 31.0% to 3.6% (31.0–27.4%).

### 4.3. The Interference Effect of the Packaging on Attention to Fat and Sodium Content Information

Seen from Table 5, the respondent’s attention to fat and sodium content information was positively influenced by health risk perception levels but negatively influenced by the interaction term between the packaging that stimulated the purchase desire and health risk perception levels. As for the marginal effect, the probability of the respondent’s attention to fat and sodium content information showed reduction in response to the packaging that stimulated the purchase desire. In particular, the probability of attention to fat content information of sauces decreased the most (by 12.6%), from 29.5% to 16.9% (29.5–12.6%) despite the health risk perception level increased by 1 point. Meanwhile, the probability of attention to sodium content information of puffed foods decreased the most (by 6.6%), from 15.6% to 9% (15.6–6.6%).

**Table 5:** Regression results of the packaging on the influence of health risk perception levels on attention to fat and sodium content information.

	Pastry Foods		Quick-Frozen Foods		Dessert Foods		Puffed Foods		**Beverages**		**Sauces**	
	Coff.	Mgn. Eff.	Coff.	Mgn. Eff.	Coff.	Mgn. Eff.	Coff.	Mgn. Eff.	**Coff.**	**Mgn.** **Eff.**	**Coff.**	**Mgn.** **Eff.**
Interference effect of the packaging on attention to fat content information												
Health risk perception levels	0.086 *** (2.85)	0.086 *** (2.85)	0.107 *** (3.92)	0.084 *** (3.30)	0.102 *** (2.92)	0.065 *** (2.54)	0.207 *** (3.59)	0.176 *** (3.02)	0.208 *** (3.02)	0.143 *** (2.73)	0.323 * (1.93)	0.295 * (1.49)
Packaging stimulates desire to buy × Health risk perception levels	−0.231 *** (−2.76)	−0.019 *** (−2.53)	−0.075 ** (−2.10)	−0.048 ** (−1.93)	−0.120 ** (−2.08)	−0.015 ** (−1.89)	−0.118 ** (−2.03)	−0.032 ** (−1.99)	−0.039 ** (−2.06)	−0.017 ** (−1.85)	−0.330 ** (−1.94)	−0.126 ** (−1.85)
Interference effect of the packaging on attention to sodium content information												
Health risk perception levels	0.152 *** (3.20)	0.072 *** (2.76)	0.083 * (1.92)	0.069 * (1.80)	0.073 ** (2.11)	0.048 ** (1.99)	0.269 *** (2.97)	0.156 *** (2.65)	0.129 * (1.70)	0.045 * (1.58)	0.194 *** (6.20)	0.154 *** (5.92)
Packaging stimulates desire to buy × Health risk perception levels	−0.126 ** (−2.17)	−0.057 ** (−2.08)	−0.058 ** (−2.12)	−0.032 ** (−2.01)	−0.039 ** (−2.12)	−0.022 ** (−2.01)	−0.085 *** (−2.64)	−0.066 *** (−2.32)	−0.019 ** (−2.09)	−0.008 ** (−1.98)	−0.059 ** (2.34)	−0.035 ** (2.01)
Control variables	Yes		Yes		Yes		Yes		Yes		Yes	
Obs.	920		920		920		920		920		920	

Source: The author’s own illustration. Note: Z statistic in parentheses; * *p* < 0.1, ** *p* < 0.05, *** *p* < 0.01.

Table 6 presents that the respondent’s levels of knowledge about fat and sodium had significantly positive impact while the interaction term between levels of knowledge about fat and sodium and the packaging that stimulated the purchase desire showed negative influence. As for the marginal effect, the probability of the respondent’s attention to fat and sodium content information increased with high level of knowledge about fat and sodium and then decreased with the intervention of the packaging that stimulated the purchase desire, especially for the dessert foods and puffed foods. The probability of the former fat content information decreased the most (by 15.9%), from 22.1% to 6.2% (22.1–15.9%) while the latter sodium content information decreased the most (by 14.3%), from 28.9% to 14.6% (28.9–14.3%).

**Table 6:** Regression results of the packaging on the influence of knowledge about fat and sodium on attention to fat and sodium content information.

	Pastry Foods		Quick-Frozen Foods		Dessert Foods		Puffed Foods		Beverages		Sauces	
	Coff.	Mgn. Eff.	Coff.	Mgn. Eff.	Coff.	Mgn. Eff.	Coff.	Mgn. Eff.	Coff.	Mgn. Eff.	Coff.	Mgn. Eff.
Interference effect of the packaging on attention to fat content information												
Levels of knowledge about fat	0.219 *** (3.77)	0.202 *** (3.53)	0.279 *** (3.63)	0.213 *** (3.14)	0.254 *** (4.298)	0.221 *** (3.46)	0.179 * (1.92)	0.102 * (1.70)	0.120 * (1.75)	0.099 * (1.65)	0.051 *** (2.92)	0.049 *** (2.41)
Packaging stimulates desire to buy × Levels of knowledge about fat	−0.210 ** (−2.08)	−0.043 ** (−1.99)	−0.165 * (−1.95)	−0.135 * (−1.61)	−0.212 * (−1.84)	−0.159 * (−1.65)	−0.139 * (−1.98)	−0.059 * (−1.63)	−0.102 ** (−1.92)	−0.079 ** (−1.88)	−0.067 * (−1.99)	−0.026 * (−1.62)
Interference effect of the packaging on attention to sodium content information												
Levels of knowledge about sodium	0.271 *** (5.13)	0.207 *** (4.88)	0.395 *** (6.99)	0.342 *** (5.60)	0.295 *** (5.09)	0.265 *** (4.82)	0.312 *** (5.61)	0.289 *** (5.02)	0.267 *** (5.32)	0.213 *** (4.02)	0.421 *** (6.85)	0.305 *** (6.27)
Packaging stimulates desire to buy × Levels of knowledge about sodium	−0.318 ** (−2.16)	−0.130 ** (−1.98)	−0.109 ** (−2.19)	−0.075 ** (−2.02)	−0.140 ** (−1.98)	−0.039 ** (−1.87)	−0.185 ** (−2.86)	−0.143 ** (−2.02)	−0.079 * (−2.08)	−0.068 * (−1.88)	−0.105 * (−1.77)	−0.065 * (−1.65)
Control variables	Yes		Yes		Yes		Yes		Yes		Yes	
Obs.	920		920		920		920		920		920	

Source: The author’s own illustration. Note: Z statistic in parentheses; * *p* < 0.1, ** *p* < 0.05, *** *p* < 0.01.

### 4.4. The Interference Effect of Flavor Labels in Subgroups

Table 7 illustrates that the weakening effect of flavor labels that stimulated the purchase desire on the positive influence of the respondent’s health risk perception levels and levels of knowledge about fat and sodium on his or her attention to fat and sodium content information differed from the classification of gender, age, income level, education level, BMI.

**Table 7:** Margin effect of flavor labels on the influence of perception and knowledge on attention to fat and sodium content information by subgroups.

	Male (*n* = 394)	Female (*n* = 526)	Youth (*n* = 554)	Middle-Aged (*n* = 366)	High-Income (*n* = 524)	Low-Income (*n* = 396)	Below College (*n* = 175)	College or Above (*n* = 745)	Not Overweight or Obese (*n* = 667)	Overweight and Obese (*n* = 253)
Interference effect of flavor labels on perception’s effect on attention to fat content information										
Pastry foods	0.119	0.009	0.096	0.120	0.086	0.062	0.008	0.091	0.122	0.140
Quick-frozen foods	0.116	0.080	0.091	0.124	0.104	0.100	0.040	0.055	0.129	0.218
Dessert foods	0.040	0.032	0.120	0.123	0.070	0.065	0.018	0.037	0.022	0.057
Puffed foods	0.176	0.120	0.167	0.194	0.100	0.097	0.085	0.164	0.181	0.186
Beverages	0.171	0.120	0.172	0.174	0.161	0.140	0.048	0.095	0.147	0.165
Sauces	0.083	0.082	0.057	0.091	0.043	0.038	0.042	0.085	0.094	0.136
Interference effect of flavor labels on perception’s effect on attention to sodium content information										
Pastry foods	0.023	0.022	0.085	0.148	0.076	0.042	0.033	0.075	0.044	0.047
Quick-frozen foods	0.073	0.009	0.004	0.006	0.013	0.001	0.068	0.078	0.060	0.071
Dessert foods	0.049	0.045	0.020	0.084	0.041	0.030	0.079	0.085	0.020	0.051
Puffed foods	0.091	0.037	0.033	0.034	0.168	0.130	0.164	0.165	0.132	0.214
Beverages	0.081	0.006	0.041	0.073	0.033	0.018	0.027	0.040	0.113	0.167
Sauces	0.184	0.166	0.082	0.086	0.093	0.086	0.136	0.147	0.115	0.172
Interference effect of flavor labels on knowledge’s effect on attention to fat content information										
Pastry foods	0.195	0.154	0.175	0.250	0.305	0.224	0.094	0.232	0.187	0.216
Quick-frozen foods	0.366	0.243	0.194	0.234	0.251	0.184	0.226	0.249	0.168	0.322
Dessert foods	0.012	0.009	0.200	0.209	0.418	0.395	0.140	0.227	0.128	0.373
Puffed foods	0.164	0.122	0.065	0.146	0.157	0.039	0.066	0.089	0.130	0.191
Beverages	0.122	0.108	0.060	0.158	0.252	0.185	0.095	0.101	0.050	0.255
Sauces	0.178	0.138	0.074	0.078	0.178	0.164	0.100	0.127	0.020	0.100
Interference effect of flavor labels on knowledge’s effect on attention to sodium content information										
Pastry foods	0.207	0.158	0.287	0.335	0.296	0.230	0.300	0.375	0.350	0.446
Quick-frozen foods	0.289	0.255	0.242	0.300	0.152	0.124	0.300	0.350	0.270	0.318
Dessert foods	0.037	0.029	0.181	0.269	0.261	0.207	0.230	0.317	0.183	0.258
Puffed foods	0.268	0.240	0.243	0.320	0.195	0.114	0.268	0.281	0.219	0.284
Beverages	0.224	0.149	0.151	0.186	0.168	0.131	0.228	0.248	0.201	0.243
Sauces	0.343	0.257	0.215	0.327	0.256	0.234	0.458	0.517	0.217	0.256

Source: The author’s own illustration. Note: marginal effect with *p* < 0.1.

In terms of the probability of the respondent’s attention to fat content information affected by both flavor labels and health risk perception levels, puffed foods, sauces, pastry foods, pastry foods, and quick-frozen foods were the UPF that showed the largest gap between females and males at 5.6% (17.6–12.0%), middle-aged people and young people at 3.4% (9.1–5.7%), high- and low-income people at 2.4% (8.6–6.2%), those who had college education and those without at 8.3% (9.1–0.8%), those who were overweight and obese, and those who were not at 8.9% (21.8–12.9%), respectively.

As for the probability of the respondent’s attention to sodium content information affected by both flavor labels and health risk perception levels, beverages, dessert foods, puffed foods, pastry foods and puffed foods were the UPF that showed the largest gap between females and males at 7.5% (8.1–0.6%), middle-aged people and young people at 6.4% (8.4–2.0%), high- and low-income people at 3.8% (16.8–13.0%), those who had college education and those without at 4.2% (7.5–3.3%), those who were overweight and obese and those who were not at 8.2% (21.4–13.2%), respectively.

With respect to the probability of the respondent’s attention to fat content information affected by both flavor labels and levels of knowledge about fat, puffed foods, beverages, puffed foods, pastry foods, and dessert foods were the UPF that showed the largest gap between females and males at 4.2% (16.94–12.2%), middle-aged people and young people at 9.8% (15.8–6.0%), high- and low-income people at 11.8% (15.7–3.9%), those who had a college education and those without at 13.8% (23.3–9.4%), and those who were overweight and obese and those who were not at 24.5% (37.3–12.8%), respectively.

As regards the probability of the respondent’s attention to sodium content information affected by both flavor labels and levels of knowledge about sodium, sauces, sauces, puffed foods, dessert foods, and pastry foods were the UPF that showed the largest gap between females and males at 8.6% (34.3–25.7%), middle-aged people and young people at 11.2% (32.7–21.5%), high- and low-income people at 8.1% (19.5–11.4%), those who had college education and those without at 8.7% (31.7–23.0%), and those who were overweight and obese and those who were not at 9.6% (44.6–35%), respectively.

### 4.5. The Interference Effect of the Packaging by Subgroups

As could be seen from Table 8, the UPF’s packaging that stimulated the purchase desire decreased the positive effect of the respondent’s health risk perception levels and levels of knowledge about fat and sodium on his or her attention to fat and sodium content information, especially in female, youth, low-income, below college, non-overweight, and obese groups.

**Table 8:** Margin effect of packaging on the influence of perception and knowledge on attention to fat and sodium content information by subgroups.

	Male (*n* = 394)	Female (*n* = 526)	Youth (*n* = 554)	Middle-Aged (*n* = 366)	High-Income (*n* = 524)	Low-Income (*n* = 396)	Below College (*n* = 175)	College or Above (*n* = 745)	**Not Overweight or Obese** **(*n* = 667)**	**Overweight and Obese** **(*n* = 253)**
Interference effect of the packaging on perception’s effect on attention to fat content information										
Pastry foods	0.106	0.080	0.106	0.174	0.147	0.126	0.098	0.107	0.090	0.097
Quick-frozen foods	0.081	0.070	0.067	0.134	0.140	0.108	0.017	0.064	0.106	0.215
Dessert foods	0.047	0.024	0.037	0.087	0.066	0.020	0.129	0.143	0.010	0.117
Puffed foods	0.176	0.100	0.186	0.203	0.123	0.108	0.160	0.173	0.047	0.116
Beverages	0.110	0.101	0.120	0.127	0.148	0.120	0.094	0.136	0.133	0.138
Sauces	0.078	0.066	0.022	0.103	0.126	0.050	0.050	0.081	0.018	0.086
Interference effect of the packaging on perception’s effect on attention to sodium content information										
Pastry foods	0.015	0.011	0.081	0.089	0.083	0.032	0.032	0.075	0.034	0.048
Quick-frozen foods	0.107	0.097	0.107	0.114	0.050	0.040	0.084	0.087	0.068	0.080
Dessert foods	0.042	0.039	0.018	0.066	0.035	0.027	0.090	0.130	0.025	0.070
Puffed foods	0.200	0.114	0.116	0.175	0.130	0.104	0.046	0.110	0.096	0.178
Beverages	0.063	0.001	0.093	0.106	0.030	0.009	0.050	0.058	0.401	0.464
Sauces	0.139	0.097	0.187	0.235	0.201	0.130	0.088	0.093	0.133	0.180
Interference effect of the packaging on knowledge’s effect on attention to fat content information										
Pastry foods	0.184	0.155	0.123	0.286	0.216	0.188	0.227	0.254	0.187	0.224
Quick-frozen foods	0.170	0.162	0.137	0.250	0.232	0.190	0.114	0.182	0.210	0.286
Dessert foods	0.193	0.185	0.207	0.223	0.165	0.151	0.209	0.212	0.147	0.246
Puffed foods	0.116	0.113	0.100	0.116	0.052	0.023	0.062	0.086	0.108	0.146
Beverages	0.217	0.152	0.120	0.138	0.214	0.150	0.167	0.206	0.117	0.128
Sauces	0.002	0.001	0.033	0.040	0.085	0.077	0.031	0.058	0.111	0.168
Interference effect of the packaging on knowledge’s effect on attention to sodium content information										
Pastry foods	0.437	0.374	0.350	0.386	0.280	0.210	0.198	0.284	0.262	0.267
Quick-frozen foods	0.384	0.310	0.284	0.310	0.177	0.163	0.288	0.295	0.024	0.028
Dessert foods	0.200	0.171	0.200	0.264	0.277	0.200	0.230	0.233	0.239	0.294
Puffed foods	0.284	0.177	0.217	0.300	0.275	0.176	0.227	0.265	0.210	0.242
Beverages	0.218	0.175	0.114	0.196	0.170	0.124	0.262	0.301	0.178	0.252
Sauces	0.267	0.230	0.219	0.330	0.232	0.186	0.217	0.263	0.275	0.297

Source: The author’s own illustration. Note: marginal effect with *p* < 0.1.

As for the probability of the respondent’s attention to fat content information affected by both the packaging and health risk perception levels, puffed foods, sauces, dessert foods, quick-frozen foods, and quick-frozen foods were the UPF that showed the largest gap between females and males at 7.6% (17.6–10%), middle-aged people, and young people at 8.1% (10.3–2.2%), high- and low-income people at 7.6% (12.6–5%), those who had college education and those without at 4.7% (6.4–1.7%), and those who were overweight and obese and those who were not at 10.9% (21.5–10.6%), respectively.

With regard to the probability of the respondent’s attention to sodium content information affected by both the packaging and health risk perception levels, puffed foods, puffed foods, sauces, puffed foods, and puffed foods were the UPF that showed the largest gap between females and males at 8.6% (20.0–11.4%), middle-aged people and young people at 5.9% (17.5–11.6%), high- and low-income people at 7.1% (20.1–13.0%), those who had college education and those without at 4.6% (11.0–4.6%), and those who were overweight and obese and those who were not at 8.2% (17.8–9.6%), respectively.

As regards the probability of the respondent’s attention to the fat content information affected by both the packaging and levels of knowledge about fat, beverages, pastry foods, beverages, quick-frozen foods, and dessert foods were the UPF that showed the largest gap between females and males at 6.5% (21.7–15.2%), middle-aged people and young people at 16.3% (28.6–12.3%), high- and low-income people at 6.4% (21.4–15.0%), those who had college education and those without at 6.8% (18.2–11.4%), and those who were overweight and obese and those who were not at 9.9% (24.6–14.7%), respectively.

In terms of the probability of the respondent’s attention to the sodium content information affected by both the packaging and levels of knowledge about sodium, puffed foods, sauces, puffed foods, pastry foods and beverages were the UPF that showed the largest gap between females and males at 10.7% (28.4–17.7%), middle-aged people and young people at 11.1% (33–21.9%), high- and low-income people at 9.9% (27.5–17.6%), those who had college education and those without at 8.6% (28.4–19.8%), and those who were overweight and obese and those who were not at 7.4% (25.2–17.8%), respectively.

## 5. Discussion

### 5.1. Discussion on the Interference Effect of Flavor Labels on Attention to Fat and Sodium Content Information

Hypotheses 1, 2, 3, and 4 were supported, suggesting that the stimulation of the consumers’ purchase desire by flavor labels reduced the probability of attention to fat and sodium content information being enhanced by the health risk perception level or the level of knowledge about fat and sodium. This finding further explained the interference effect of purchase desire stimulated by flavor labels described in the theoretical framework for ultra-processed food purchasing decisions. Flavor labels indeed played a weakening role rather than counteracting role in the promoting effect of the level of health risk perception and knowledge. In other words, consumers having high level of health risk perception and knowledge were still helpful for attention to fat and sodium information despite of the influence of flavor labels. However, the largest decline in consumers’ attention to fat and sodium content information of dessert foods and puffed foods was due to the fact that food manufacturers put exciting flavor labels such as chocolate-flavored ice cream and roast meat-flavored potato chips on these foods [32]. Even though consumers knew that the excessive consumption of dessert foods and puffed foods increases the risk of suffering from chronic disease, they showed the greatest reduction in the attention they pay to fat and sodium content information. Meanwhile, the largest decline in consumers’ attention to fat and sodium content information of puffed foods and quick-frozen foods was due to the fact that these foods had flavor labels that made people feel good, such as chocolate-flavored ice cream and roast meat-flavored potato chips [53]. Even if consumers had knowledge about fat and sodium—that is, they knew that the excessive intake of fat and sodium each day increases the risk of obesity and high blood pressure—they paid less attention.

### 5.2. Discussion on the Interference Effect of the Packaging on Attention to Fat and Sodium Content Information

The findings supported Hypotheses 5, 6, 7, and 8. As the stimulation of consumers’ purchase desire by the packaging of UPF was proved to reduce the probability of attention to fat and sodium content information being enhanced by the level of health risk perception or knowledge about fat and sodium. Still, it was observed that the packaging generated the weakening effect, which further explained the interference effect of purchase desire stimulated by flavor labels described in the theoretical framework for ultra-processed food purchasing decisions. This reflected that Chinese consumers’ rational cognition such as the high level of health risk perception and knowledge triumphed over their emotional response such as desire to buy stimulated by flavor labels and the packaging. The reason sauces and puffed foods showed the greatest negative impact in terms of consumers with health risk perception paying attention to fat and sodium content information is that there were celebrity endorsements, bright colors, and exquisite patterns on the packaging of sauces and puffed foods, which generated an obvious visual impact and made consumers choose the foods without hesitation. And the reason dessert foods and pastry foods showed the greatest negative impact in terms of consumers with knowledge about fat and sodium paying attention to fat and sodium content information was that the packaging of these products was generally beautiful and colorful, and it was easy for consumers to make impulsive purchase decisions.

### 5.3. Discussion on the Interference Effect of Flavor Labels by Subgroup

Hypotheses 9 and 10 were supported, revealing that the stimulation of consumers’ purchase desire by flavor labels weakened the enhancement of their attention to fat and sodium content information by high level of health risk perception and knowledge about fat or sodium, with significant population differences. In particular, females, youth, low-income individuals, those with below-college education, and non-overweight/non-obese individuals paid less attention to the information. This provides a reference for the authority to carry out nutrition labeling education for key subgroups. Specifically speaking, women tended to be more sensitive to and easily affected by flavor labels of beverages when shopping, leading to less attention being given to the information than men; people with below-college education tended to have lower cultural literacy and less understanding of the relationship between the flavor of UPF and health risks, and paid less attention to fat and sodium content information of pastry foods than those with college education or above. In addition, the probability of attention to fat and sodium content information of puffed foods showed the largest gap between high-and low-income people. This was because puffed foods were moderately priced and relatively economic choices for low-income people. There were a wide variety of puffed foods with original and various other flavors, and low-income people could enrich their diets by choosing different flavors of puffed foods [54]. Even if low-income people had the knowledge, the flavor labels of puffed foods may induce them to pay less attention to the health information and make purchasing decisions quickly.

### 5.4. Discussion on the Interference Effect of Packaging by Subgroup

Hypotheses 11 and 12 were supported, revealing that the stimulation of consumers’ purchase desire by the packaging weakened the enhancement of their attention to fat and sodium content information by having health risk perception and knowledge about fat and sodium, with significant population differences, especially females, youth, low-income individuals, those with below-college education, and non-overweight/non-obese individuals paid less attention. This was because the colors and patterns on the front of the UPF packaging made consumers ignore the information during purchase and lead them to immerse themselves in visual enjoyment, influencing their purchase decisions. In terms of the impact of the packaging, the probability of consumers with health risk perception paying increased attention to fat and sodium content information of puffed foods showed the largest gap between males and females. This was because the unique, interesting, and fashionable puffed food packaging could attract females’ attention and trigger their purchase desire.

### 5.5. Limitations

Several limitations need to be noted. Firstly, self-report questionnaires were used to collect data on respondents’ emotional response and rational behavior, resulting in subjective data. Instead, future research should collect non-self-reported data using experimental methods that simulate real shopping scenes. Secondly, this study failed to measure emotional responses with discount promotions because cheaper foods were more attractive; the effect of discount labels could be studied. Thirdly, illness experience was not used to measure respondents’ rational cognition, even though chronic nutritional disease caused by the excessive consumption of UPF may increase the probability of attention to fat and sodium content information, which may have caused the regression estimation results to be overestimated. Fourthly, there was a lack of sub-population analysis of the proportion of households under 18 years old and 60 years old and above, because consumers bought foods based on a consideration of the healthiness of the diets of minors and elderly people in the family, increasing their attention to nutrition labeling information. Finally, the only nutritional aspects of the foods investigated in this study were fat and sodium. Therefore, it is not known how flavor labels and the packaging might affect the attention paid by consumers to other health-related aspects of the foods such as the content of fibre and energy.

## 6. Conclusions

This study arrived at the following conclusions: (1) The stimulation of consumers’ desire to buy UPF by flavor labels reduced the probability of their attention to fat and sodium content information being enhanced by health risk perception and knowledge about fat and sodium; in particular, the attention probability for dessert foods, puffed foods, and quick-frozen foods decreased the most. (2) The stimulation of consumers’ desire to buy UPF by the packaging reduced the probability of their attention to fat and sodium content information being enhanced by health risk perception and knowledge about fat and sodium; in particular, the attention probability for sauces, puffed foods, dessert foods, and pastry foods decreased the most. (3) Compared with other groups, females, youth, low-income individuals, those with below-college education, and non-overweight/non-obese individuals whose desire to buy UPF was stimulated by flavor labels showed a reduced probability of their attention to fat and sodium content information being enhanced by health risk perception and knowledge about fat and sodium; in particular, beverages, pastry foods, and puffed foods showed the largest differences in attention among the different groups. (4) Females, youth, low-income individuals, those with below-college education, and non-overweight/non-obese individuals whose desire to buy UPF was stimulated by flavor labels showed a further reduced probability of their attention to fat and sodium content information being enhanced by health risk perception and knowledge about fat and sodium; in particular, puffed foods showed the largest difference in attention among the different groups.

Some policy recommendations are offered. First, consumers need to be reminded not to make purchasing decisions only based on flavor labels and the packaging of UPF, but also to take these foods’ fat and sodium content information into consideration. Second, public nutrition and health education should be strengthened, and consumers’ health risk perception of UPF and knowledge about sodium and fat should be improved. These recommendations apply not only to the fat and sodium content of the foods but also to the overall nutritional value of food. Finally, it is essential to attach importance to and improve the nutritional literacy of females, young people, low-income individuals, those with below-college education, and non-overweight/non-obese individuals.

## Figures and Tables

**Table 2:** Variables’ descriptions and summary statistics.

Variable	Definition	Mean	Std.Dev.	Min.	Max.	Percentage %	Obs.
Dependent variable							
Attention to fat content information on pastry foods	No	—	—	—	—	48.9	450
	Yes	—	—	—	—	51.1	470
Attention to sodium content information on pastry foods	No	—	—	—	—	74.0	681
	Yes	—	—	—	—	26.0	239
Attention to fat content information on quick-frozen foods	No	—	—	—	—	50.7	466
	Yes	—	—	—	—	49.4	454
Attention to sodium content information on quick-frozen foods	No	—	—	—	—	68.6	631
	Yes	—	—	—	—	31.4	289
Attention to fat content information on dessert foods	No	—	—	—	—	59.9	551
	Yes	—	—	—	—	40.1	369
Attention to sodium content information on dessert foods	No	—	—	—	—	73.2	673
	Yes	—	—	—	—	26.9	247
Attention to fat content information on puffed foods	No	—	—	—	—	54.6	502
	Yes	—	—	—	—	45.4	418
Attention to sodium content information on puffed foods	No	—	—	—	—	70.4	648
	Yes	—	—	—	—	29.6	272
Attention to fat content information on beverages	No	—	—	—	—	64.1	590
	Yes	—	—	—	—	35.9	330
Attention to sodium content information on beverages	No	—	—	—	—	75.2	692
	Yes	—	—	—	—	24.8	228
Attention to fat content information on sauces	No	—	—	—	—	72.2	664
	Yes	—	—	—	—	27.8	256
Attention to sodium content information on sauces	No	—	—	—	—	57.6	530
	Yes	—	—	—	—	42.4	390
Independent variable							
Levels of knowledge about fat	Score	3.48	0.64	1	4	—	920
Levels of knowledge about sodium	Score	3.24	0.97	1	4	—	920
Health risk perception levels regarding pastry foods	Score	3.98	0.79	1	5	—	920
Health risk perception levels regarding quick-frozen foods	Score	4.21	0.98	1	5	—	920
Health risk perception levels regarding dessert foods	Score	3.77	0.62	1	5	—	920
Health risk perception levels regarding puffed foods	Score	4.10	0.75	1	5	—	920
Health risk perception levels regarding beverages	Score	4.41	0.84	1	5	—	920
Health risk perception levels regarding sauces	Score	3.36	0.63	1	5	—	920
Control variable							
	Female	—	—	—	—	57.2	526
	Male	—	—	—	—	42.8	394
Age	Years	35.46	9.35	18	58	—	920
Education level	Primary school or below	—	—	—	—	0.43	4
	Junior school	—	—	—	—	4.67	43
	Senior school	—	—	—	—	13.9	128
	College level	—	—	—	—	73.7	678
	Postgraduate or above	—	—	—	—	7.28	67
Annual household disposable income (CNY ^a^)	<10,000	—	—	—	—	6.63	61
	10,000~50,000	—	—	—	—	15.8	145
	50,001~100,000	—	—	—	—	20.5	189
	100,001~150,000	—	—	—	—	24.0	221
	150,001~200,000	—	—	—	—	19.6	180
	≥200,001	—	—	—	—	13.5	124
Minors in households	%	0.24	0.16	0	1.5	—	920
Elderly people in households	%	0.18	0.24	0	2	—	920
Suffer from chronic diseases	No	—	—	—	—	72.4	666
	Yes	—	—	—	—	27.6	254
Comprehensible NFT information	No	—	—	—	—	77.5	713
	Yes	—	—	—	—	22.5	207
Accurate NFT information	No	—	—	—	—	61.0	561
	Yes	—	—	—	—	39.0	359
Authoritative NFT information	No	—	—	—	—	39.8	366
	Yes	—	—	—	—	60.2	554
Flavor labels stimulate desire to buy	No	—	—	—	—	18.0	166
	Yes	—	—	—	—	82.0	754
The packaging stimulates desire to buy	No	—	—	—	—	36.9	339
	Yes	—	—	—	—	63.2	581

Source: The author’s own illustration. Note: ^a^ USD 1 equals CNY 7.11, and EUR 1 equals CNY 7.74 from 10 October to 25 October 2024.

**Table 3:** Regression results of flavor labels on the influence of health risk perception levels on attention to fat and sodium content information.

	Pastry Foods		Quick-Frozen Foods		Dessert Foods		Puffed Foods		Beverages		Sauces	
	Coff.	Mgn. Eff.	Coff.	Mgn. Eff.	Coff.	Mgn. Eff.	Coff.	Mgn. Eff.	Coff.	Mgn. Eff.	Coff.	Mgn. Eff.
Interference effect of flavor labels on attention to fat content information												
Health risk perception levels	0.237 *** (2.93)	0.110 *** (2.28)	0.208 ** (2.81)	0.113 ** (2.56)	0.288 *** (3.08)	0.131 *** (2.71)	0.355 *** (4.13)	0.142 *** (3.85)	0.271 *** (2.80)	0.146 *** (2.28)	0.132 ** (2.94)	0.085 ** (2.00)
Flavor labels stimulate desire to buy × health risk perception levels	−0.138 ** (−2.78)	−0.042 ** (−2.21)	−0.170 ** (−2.05)	−0.012 ** (−1.89)	−0.123 ** (−2.46)	−0.096 ** (−2.02)	−0.175 ** (−2.04)	−0.086 ** (−1.97)	−0.122 ** (−2.03)	−0.005 ** (−1.88)	−0.121 ** (−2.58)	−0.075 ** (−2.02)
Interference effect of flavor labels on attention to sodium content information												
Health risk perception levels	0.133 *** (3.10)	0.079 *** (2.82)	0.127 ** (2.22)	0.053 ** (2.03)	0.210 *** (3.04)	0.055 *** (2.68)	0.200 *** (2.81)	0.144 *** (2.43)	0.049 ** (2.05)	0.031 ** (1.80)	0.192 *** (5.99)	0.178 *** (5.68)
Flavor labels stimulate desire to buy × health risk perception levels	−0.121 *** (−3.01)	−0.046 *** (−2.45)	−0.101 ** (−2.16)	−0.028 ** (−1.99)	−0.120 ** (−2.03)	−0.053 ** (−1.99)	−0.187 ** (−2.54)	−0.108 ** (−2.30)	−0.237 ** (−2.05)	−0.022 ** (−1.98)	−0.082 ** (2.07)	−0.051 ** (1.95)
Control variables	Yes		Yes		Yes		Yes		Yes		Yes	
Obs.	920		920		920		920		920		920	

Source: The author’s own illustration. Note: Z statistic in parentheses; ** *p* < 0.05, *** *p* < 0.01.

**Table 4:** Regression results of flavor labels on the influence of levels of knowledge about fat and sodium on attention to fat and sodium content information.

	Pastry Foods		Quick-Frozen Foods		Dessert Foods		Puffed Foods		Beverages		Sauces	
	Coff.	Mgn. Eff.	Coff.	Mgn. Eff.	Coff.	Mgn. Eff.	Coff.	Mgn. Eff.	Coff.	Mgn. Eff.	Coff.	Mgn. Eff.
Interference effect of flavor labels on attention to fat content information												
Levels of knowledge about fat	0.365 *** (3.84)	0.240 *** (3.40)	0.252 *** (3.90)	0.231 *** (3.46)	0.195 *** (2.70)	0.168 *** (2.54)	0.320 * (1.99)	0.205 * (1.70)	0.158 ** (2.77)	0.139 ** (2.04)	0.216 ** (2.22)	0.132 ** (2.07)
Flavor labels stimulate desire to buy × Levels of knowledge about fat	−0.203 *** (−4.02)	−0.142 *** (−3.88)	−0.142 * (−2.05)	−0.109 * (−1.48)	−0.048 ** (−2.13)	−0.035 ** (−1.94)	−0.193** (−2.35)	−0.176 ** (−2.00)	−0.059 ** (−2.06)	−0.040 ** (−1.95)	−0.152 * (−1.95)	−0.038 * (−1.75)
Interference effect of flavor labels on attention to sodium content information												
Levels of knowledge about sodium	0.318 *** (4.95)	0.209 *** (4.02)	0.374 *** (4.74)	0.310 *** (4.42)	0.299 ** (5.17)	0.286 ** (4.56)	0.370 *** (4.92)	0.328 *** (4.36)	0.198 *** (3.82)	0.186 *** (3.65)	0.384 *** (5.95)	0.301 *** (5.75)
Flavor labels stimulate desire to buy × levels of knowledge about sodium	−0.307 * (−1.79)	−0.266 * (−1.67)	−0.372 ** (−2.29)	−0.274 ** (−2.12)	−0.179 ** (−1.99)	−0.131 ** (−1.87)	−0.184 * (−1.97)	−0.168 * (−1.85)	−0.051 ** (−2.05)	−0.036 ** (−1.85)	−0.144 ** (−1.96)	−0.053 ** (−1.85)
Control variables	Yes	Yes	Yes	Yes			Yes			Yes		
Obs.	920		920		920		920		920		920	

Source: The author’s own illustration. Note: Z statistic in parentheses; * *p* < 0.1, ** *p* < 0.05, *** *p* < 0.01.

## Data Availability

The original contributions presented in the study are included in the article, further inquiries can be directed to the corresponding author.

## Supplementary Materials

The following supporting information can be downloaded at: www.mdpi.com/article/10.3390/nu17010174/s1. The questionnaires are available online.
