# Supplementary material for: Reason’s Triumph over Passion? Chinese Adults’ Attention to Information on Ultra-Processed Foods’ Fat and Sodium Contents in Nutrition Facts Tables
Source: Nutrients. 2025 Jan 2;17(1):174. doi: 10.3390/nu17010174 (PMC11722638; doi:10.3390/nu17010174)
Supplement: Supplementary file 1 [file nutrients-17-00174-s001.zip › nutrients-3360969-supplementary.pdf]

The questionnaire

Section 1 Demographic characteristics

Q1-1 Your gender: A. Male B. Female

Q1-2 Your birth year: ______

Q1-3 Your height: ______cm

Q1-4 Your weight: ______kg

Q1-5 Your education level: A. Primary school or below B. Junior school C. Senior school D. Junior college or undergraduate E. Postgraduate or above

Q1-6 Your annual personal disposable income : ______Chinese Yuan

A.Less than 10,000 yuan

B.10,000～50,000 yuan

C.50,001～100,000 yuan

D.100,001～150,000 yuan

E.150,001～200,000 yuan

F.200,001 yuan and above

Q1-7 There are: ______ permanent residents in your family. Among them, there are: ______residents under the age of 18 and ______residents aged 60 and over.

Q1-8 Have you suffered from nutrition-related chronic diseases such as high blood pressure, diabetes, hyperlipidemia or obesity? A.Yes B. No

Section 2 Nutrition knowledge level

The following is a three-question knowledge quiz about sodium.

Q2-1 What is the main effect of sodium on the human body?

A. Regulates fluid balance B. Antioxidants C. Contributes to bone health D. Helps skin health

Q2-2 What is the maximum daily intake of sodium for an normal adult?

A.1000 mg B.1500 mg C.2000 mg D.2500 mg

Q2-3 What are the main diseases caused by excessive intake of sodium?

A. Hypertension B. Obesity C. Hyperlipidemia D. Diabetes

The following is a three-question knowledge quiz about fat.

Q2-4 What is the main function of fat on the human body?

A.Maintains normal intestinal function B. Promotes bone formation

C. Helps absorb fat-soluble vitamins D. Antioxidant

Q2-5 What is the maximum daily intake of fat for an normal adult?

A.60 grams B.100 grams C.140 grams D.180 grams

Q2-6 What kind of disease does high intake of fat cause?

A. Hypertension B. Keshan disease C. Hyperlipidemia D. Night blindness

Section 3 Attitudes and behavior towards nutrition facts table

Figure 1 shows the nutrition facts table of a certain prepackaged food. Please answer the following questions.

Q3-1 I think the information on the nutrition facts table is comprehensible. A.Yes B. No

Q3-2 I think the information on the nutrition facts table is accurate. A.Yes B. No

Q3-3 I think the information on the nutrition facts table is authoritative. A.Yes B. No

Q3-4 Do you pay attention to fat content information of ultra-processed pastry food (*e.g.,* bread, cake, cookies, moon cakes) on the nutrition facts table when shopping.
A.Yes B. No
Q3-5 Do you pay attention to sodium content information of ultra-processed pastry food (*e.g.,* Bread, cake, cookies, moon cakes) on the nutrition facts table when shopping.
A.Yes B. No
Q3-6 Do you pay attention to fat content information of ultra-processed quick-frozen food (*e.g.,* frozen pizza, frozen dumplings) on the nutrition facts table when shopping.
A.Yes B. No
Q3-7 Do you pay attention to sodium content information of ultra-processed quick-frozen food (*e.g.,* frozen pizza, frozen dumplings) on the nutrition facts table when shopping.
A.Yes B. No
Q3-8 Do you pay attention to fat content information of ultra-processed dessert food (*e.g.* chocolate, ice cream) on the nutrition facts table when shopping.
A.Yes B. No
Q3-9 Do you pay attention to sodium content information of ultra-processed dessert food (*e.g.* chocolate, ice cream) on the nutrition facts table when shopping.
A.Yes B. No
Q3-10 Do you pay attention to fat content information of ultra-processed puffed food (e.g. potato chips, French fries, shrimp sticks) on the nutrition facts table when shopping.
A.Yes B. No
Q3-11 Do you pay attention to sodium content information of ultra-processed puffed food (e.g. potato chips, French fries, shrimp sticks) on the nutrition facts table when shopping.
A.Yes B. No
Q3-12 Do you pay attention to fat content information of ultra-processed beverages (*e.g.* milk tea, soda, processed juice) on the nutrition facts table when shopping.
A.Yes B. No
Q3-13 Do you pay attention to sodium content information of ultra-processed beverages (*e.g.* milk tea, soda, processed juice) on the nutrition facts table when shopping.
A.Yes B. No
Q3-14 Do you pay attention to fat content information of ultra-processed sauces (e.g. salad dressings, mayonnaise, ketchup) on the nutrition facts table when shopping.
A.Yes B. No
Q3-15 Do you pay attention to sodium content information of ultra-processed sauces (e.g. salad dressings, mayonnaise, ketchup) on the nutrition facts table when shopping.
A.Yes B. No

Nutrition facts table

Items	Per 100 g	NRV%
Energy	2505 kJ	30
Protein	27.0 g	45
Fat	50.2 g	84
Carbohydrate	16.5 g	6
Sodium	618 mg	31

Figure S1. A nutrition facts table of a certain prepackaged food

Section 4 Attitudes towards ultra-processed foods' flavor labels and packaging.

The following is a five-question quiz about your attitude towards ultra-processed foods' flavor labels

Q4-1 Classic flavor (*e.g.* sour, spicy, salty, sweet) labels could inspire my desire to buy the food.

A.Yes B. No

Q4-2 Local flavor (*e.g.* sour and hot) labels could inspire my desire to buy the food.

A.Yes B. No

Q4-3 Exotic and unique flavor (*e.g.* Mexican barbecue) labels could inspire my desire to buy the food.

A.Yes B. No

Q4-4 Innovative flavor (*e.g.* floral and herbal) labels could inspire my desire to buy the food.

A.Yes B. No

Q4-5 Fusion flavor (*e.g.* Chinese and western) labels could inspire my desire to buy the food.

A.Yes B. No

The following is a five-question quiz about your attitude towards ultra-processed foods' packaging

Q4-6 The bright colors of packaging could inspire my desire to buy the food.

A.Yes B. No

Q4-7 The unique shape of packaging could inspire my desire to buy the food.

A.Yes B. No

Q4-8 The high quality of packaging could inspire my desire to buy the food.

A.Yes B. No

Q4-9 The beautiful pattern of packaging could inspire my desire to buy the food.

A.Yes B. No

Q4-10 The infectious copy of packaging could inspire my desire to buy the food.

A.Yes B. No

Section 5 Health risk perception of ultra-processed foods

The following is a four-question quiz about your health risk perception of ultra-processed pastry food (*e.g.,* bread, cake, cookies, moon cakes)

Q5-1 I think excessive consumption of ultra-processed pastry food could increase the risk of obesity.

A.Yes B. No

Q5-2 I think excessive consumption of ultra-processed pastry food could affect the stability of blood pressure.

A.Yes B. No
Q5-3 I think excessive consumption of ultra-processed pastry food could increase the risk of dyslipidemia.
A.Yes B. No
Q5-4 I think excessive consumption of ultra-processed pastry food could increase the risk of type 2 diabetes.
A.Yes B. No

The following is a four-question quiz about your health risk perception of ultra-processed quick-frozen food (*e.g.,* frozen pizza, frozen dumplings)
Q5-5 I think excessive consumption of ultra-processed frozen food could increase the risk of obesity.
A.Yes B. No
Q5-6 I think excessive consumption of ultra-processed frozen food could affect the stability of blood pressure.
A.Yes B. No
Q5-7 I think excessive consumption of ultra-processed frozen food could increase the risk of dyslipidemia.
A.Yes B. No
Q5-8 I think excessive consumption of ultra-processed frozen food could increase the risk of type 2 diabetes.
A.Yes B. No

The following is a four-question quiz about your health risk perception of ultra-processed dessert food (*e.g.* chocolate, ice cream)
Q5-9 I think excessive consumption of ultra-processed dessert food could increase the risk of obesity.
A.Yes B. No
Q5-10 I think excessive consumption of ultra-processed dessert food could affect the stability of blood pressure.
A.Yes B. No
Q5-11 I think excessive consumption of ultra-processed dessert food could increase the risk of dyslipidemia.
A.Yes B. No
Q5-12 I think excessive consumption of ultra-processed dessert food could increase the risk of type 2 diabetes.
A.Yes B. No

The following is a four-question quiz about your health risk perception of ultra-processed puffed food (*e.g.* potato chips, French fries, shrimp sticks)
Q5-13 I think excessive consumption of ultra-processed puffed food could increase the risk of obesity.
A.Yes B. No
Q5-14 I think excessive consumption of ultra-processed puffed food could affect the stability of blood pressure.
A.Yes B. No
Q5-15 I think excessive consumption of ultra-processed puffed food could increase the risk of

dyslipidemia.

A.Yes B. No

Q5-16 I think excessive consumption of ultra-processed puffed food could increase the risk of type 2 diabetes.

A.Yes B. No

The following is a four-question quiz about your health risk perception of ultra-processed beverages (*e.g.* milk tea, soda, processed juice)

Q5-17 I think excessive consumption of ultra-processed beverages could increase the risk of obesity.

A.Yes B. No

Q5-18 I think excessive consumption of ultra-processed beverages could affect the stability of blood pressure.

A.Yes B. No

Q5-19 I think excessive consumption of ultra-processed beverages could increase the risk of dyslipidemia.

A.Yes B. No

Q5-20 I think excessive consumption of ultra-processed beverages could increase the risk of type 2 diabetes.

A.Yes B. No

The following is a four-question quiz about your health risk perception of ultra-processed sauces (*e.g.* salad dressings, mayonnaise, ketchup)

Q5-21 I think excessive consumption of ultra-processed sauces could increase the risk of obesity.

A.Yes B. No

Q5-22 I think excessive consumption of ultra-processed sauces could destabilize blood pressure.

A.Yes B. No

Q5-23 I think excessive consumption of ultra-processed sauces could increase the risk of dyslipidemia.

A.Yes B. No

Q5-24 I think excessive consumption of ultra-processed sauces could increase the risk of type 2 diabetes.

A.Yes B. No
